# Atlanta Society of Medicine

**Published:** 1890-02

**Authors:** 


### ATLANTA SOCIETY OF MEDICINE.

W. P. Nicolson, M. D., President.

### A CASE OF LITHOTOMY.

Dr. W. S. Elkin reported a case of remov al by the lateral operation of a peculiar L shaped stone from the bladder of a boy aged 11 years. The patient had suffered with the usual symptoms of stone for preceding three years. The stone weighed 420 grs. Patient made an excellent recovery.

Dr. Baird said that he had recently been struck with the fact that a pulsating artery could be felt in front of cervix in a number of early pregnancies under his observation, and thought the sign might be one of some importance in making a diagnosis of pregnancy. He had not seen any mention made of it in obstetrical works.

Dr. Hardon, in discussing Dr. Baird's cases, said that Lusk and others recognized arterial pulsating in front of the cervix as one of the minor signs of pregnancy, and that he had been in the habit of mentioning it in his lectures. He did not consider the sign reliable as it might accompany pelvic congestion from any cause.

The following case was reported by Dr. J. G. Earnest. In July last he and Dr. R. B. Ridley were called in consultation by Dr. Goldsmith, of this city, to see a young lady in labor with her first child. The head was found presenting high up in the pelvis. Active labor had been going on for thirty-six hours and the membranes had ruptured several hours before we saw her.

The head was prevented from descending by a hard mass, the size of an orange, lying immediately below the well dilated os uteri. This mass was outside of the vagina and was lying a little to the left of the median line at the back of the vagina. It was found to be impossible to displace the head of the fœtus in such a way as to push the tumor above it. So it was determined to apply the forceps to the child and see if it could not be slowly dragged down far enough to fall into the space between the coccyx and ischium until the head would pass. This scheme was put into execution and the child safely delivered. Immediately the tumor went up with a jerk and was afterward found lying above the uterus and a little to the left. The mother and child did well for six days, when the mother developed a case of puerperal insanity. The case proving obstinate she was placed in an asylum as soon as it was thought prudent for her to make the journey. A few weeks later she was returned to her home entirely restored. The tumor was found to be but little changed, and was diagnosed as an ovarian cyst and immediate removal advised. January 4th, I operated, assisted by Doctors McCaulay and Huzza. The tumor proved to be a dermoid cyst of the left ovary the size of a medium orange. When opened a solid mass, the size of an English walnut, occupied the seat of the ovary. In this was imbedded two bicusped teeth of usual size and shape, and from it sprang a mass of hair that had grown until the whole interior of the cyst was tightly packed with it. It was saturated with the usual chyle like fluid. The patient recovered promptly.

Dr. Hardon reported a case of laparotomy and exhibited specimen of solid tumor of the ovary.

Dr. Duncan reported a case of enteritis preceded for twenty-four hours by intense pain between the knee and ankle. He considered the pain of reflex origin.

Dr. Cooper reported a case of fracture of the femur from a very unusual cause: the patient, a strong well-developed boy of fourteen, was walking very rapidly on smooth level ground; he stopped suddenly in his tracks, at the same time twisting his body half round to look at a boy, who was following him; as he did so, he felt his left hip give way, and he fell helpless to the ground.

He was picked up and taken into the house, and the doctor summoned. After etherizing the patient, examination revealed a transverse fracture of the femur just below the trochanter, with marked shortening and great deformity. With the assistance of Drs. Harris and Jones the deformity was overcome, and a plaster of Paris splint applied from the foot to the umbilicus.

The case is of interest because of the slight degree of violence which produced the fracture. In old persons fracture of the neck of the femur very commonly results from the application of trivial violence. This is entirely dependent on senile changes in the structure of the bone, and the inclination of the neck to the shaft.

In young people, however, it is very unusual to see a fracture of the femur from such an insufficient cause. The history of the boy furnished a rational explanation of the accident; for the past three years he has suffered from pain in the hip, supposed to be rheumatic; this pain was not constant, being a great deal worse in winter than in summer. Frequently for a month or two at a time the pain was sufficient to make him walk with quite a perceptible limp.

It is very reasonable to believe that this pain was caused by a chronic inflammatory process in the upper end of the femur, possibly of a tubercular character. This slow inflammation resulted in osteoporosis, a condition in which the interstitial spaces of the cancellous structure are increased in size, thereby weakening the bone. This condition being present, a very slight violence would produce a fracture.

He also reported a case of amputation of the breast for malignant disease. The patient was a middle aged lady, who had had for nearly thirty years an adenoma of the left breast, which caused her no inconvenience whatever. About six months ago the breast became the seat of severe neuralgic pains, and a gradual retraction of the nipple took place. The doctor was first consulted by her last October; at that time the tumor was about the size of a small egg, hard and dense, the nipple much retracted, and the skin around it adherent and slightly ulcerated; no involvement of the axillary glands.

Immediate removal was urged, but the patient postponed operation until ten days ago, when, with the assistance of Drs. Elkin and Harris, he removed the breast. The highest temperature the patient had after the operation was 100 F.

This case illustrates the tendency of benign growths of the breast to undergo malignant transformation.

Dr. Gaston reported a case of perineal section for the relief of stricture complicated by perineal abscess and chronic cystitis. Patient was making excellent recovery.

Dr. McRae reported a case of unusually large umbilical cord, with death 48 hours after birth from umbilical hemorrhage. Recognizing the danger from hemorrhage he had ligatured the cord with great care.

Also a case of craniotomy. Mrs. P. æt, 25, primipara was seen in consultation. Had been in labor four days and nights and was very much exausted. On making examination under anæsthesia, found pelvis contracted to about 2 inches antero-posterior measurement, vertex firmly wedged in transverse diameter of brim with vagina much lacerated and contused from ineffectual attempts at delivery with the forceps. On account of the weakened condition of the patient, Cæsarean section was deemed too hazardous and craniotomy was successfully performed in the usual way, with the strictest antiseptic precautions. The uterus and vagina were thoroughly washed out before and after delivery with a 2.5 per cent. solution or emulsion of creolin in water. The external genitals of patient were washed with a 1.1000 solution of bichloride of mercury, all soiled clothing, etc., removed and antiseptic pad applied. The dressings were changed twice daily, always with strict antiseptic precautions. At no time after operation did the temperature exceed 99.5° F. and there were absolutely no symptoms of septic absorption.

The doctor called attention to the advantages of creolin in obstetric practice, as set forth by Garrigues and others. 1st. It was unirritating. 2d. Its property of making surfaces slippery. 3d. It was an excellent hæmostatic.

Dr. Nicolson reported two cases in which the subsequent history had been followed after operation for malignant growths. In one he had one year since amputated the thigh in the upper third, for osteosarcoma of the femur, involving the bone as far as the middle. The patient made a rapid recovery, and was back at his home in a neighboring State on the twenty-first day from the time he left. The subsequent history was, that he apparently did well for some time; the growth never returned in the bone; but after an illness, covering a comparatively short time, he died a few days since from secondary deposits in the lungs.

In the second case, the patient was operated upon four years since for extensive epithelioma of the penis. There was such involvement of the lymphatic glands of both groins that it was only anticipated that the relief would be of a temporary nature. The operation was made with the paquelin cautery, the penis being supported with an ivory staff introduced into the urethra, and the tissues burned through at a red heat. As an anæsthetic, cocaine was successfully employed. There was no undue contraction of the urethral orifice, and the recovery was excellent. Recent report from him was to the effect that the glandular involvement had disappeared, and that he has been in perfect health, with no prospect of return. The remaining stump was pronounced eminently satisfactory and useful. About one inch of the organ was left.

